# Recurrence of microfilaraemia after triple-drug therapy for lymphatic filariasis in Samoa: Recrudescence or reinfection?

**DOI:** 10.1016/j.ijid.2025.107809

**Published:** 2025-03

**Authors:** Helen J. Mayfield, Ramona Muttucumaru, Benn Sartorius, Sarah Sheridan, Selina Ward, Beatris Mario Martin, Shannon M. Hedtke, Robert Thomsen, Satupaitea Viali, Glen Fatupaito, Colleen L. Lau, Patricia M. Graves

**Affiliations:** 1 University of Queensland Centre for Clinical Research, Faculty of Health, Medicine and Behavioural Sciences, University of Queensland, Brisbane, Queensland, Australia; 2 School of Public Health, Faculty of Health, Medicine and Behavioural Sciences, The University of Queensland, Brisbane, Queensland, Australia; 3 National Centre for Epidemiology and Population Health, The Australian National University, Canberra, Australia; 4 Department of Environment and Genetics, La Trobe University, Bundoora, Victoria, Australia; 5 Samoa Ministry of Health, Apia, Samoa; 6 Oceania University of Medicine Samoa, Apia, Samoa; 7 College of Public Health, Medical and Veterinary Sciences, James Cook University, Townsville, Queensland, Australia

**Keywords:** Neglected tropical diseases, Lymphatic filariasis elimination, Mass drug administration, Ivermectin, Diethylcarbamazine, Albendazole

## Abstract

**Objectives:**

Contrasting evidence is emerging on the long-term effectiveness of triple-drug therapy for elimination of lymphatic filariasis (LF) in the Pacific region. We evaluated the effectiveness of ivermectin, diethylcarbamazine and albendazole (IDA) for sustained clearance of microfilariae (Mf) in Samoa.

**Methods:**

We enrolled two cohorts of Mf-positive participants. Cohort A were Mf-positive participants from 2018, who received directly observed triple-drug therapy in 2019 and were retested and retreated in 2023 and 2024. Cohort B were Mf-positive and treated in 2023 and retested in 2024. Participants were tested for LF antigen and Mf.

**Results:**

In Cohort A, eight of the 14 participants from 2018/2019 were recruited in 2023; six were Mf-positive. In 2024, six participants were retested, and two were Mf-positive. Cohort B included eight participants, and two remained Mf-positive in 2024. Mf prevalence in 2023 for Cohort A (71.4%, 95% CI 29.0%-96.3%) was significantly higher than among their household members (12.0%, 95% CI 2.5%-31.2%).

**Conclusion:**

One or two doses of directly observed IDA was not sufficient for sustained clearance of *Wuchereria bancrofti* Mf in Samoa. The high Mf prevalence in treated individuals compared to household members suggests recrudescence rather than reinfection.

## Highlights

• Triple-drug therapy (IDA) evaluated for lymphatic filariasis (LF) elimination.
• Microfilaria (Mf) density decreased significantly 18 months post-treatment in Samoa.
• One or two doses of IDA was not sufficient for sustained Mf clearance in Samoa.
• Higher Mf prevalence in the cohort than in household members suggests recrudescence.
• Further work needed to assess the effectiveness of IDA for LF elimination in Samoa.

## Introduction

Lymphatic filariasis (LF) is a vector-borne disease which remains endemic in many regions of the world despite global elimination efforts [1]. The disease is caused by infection from a helminth parasite (*Wuchereria bancrofti, Brugia malayi* or *Brugia timori*) and can cause severe and irreversible lymphedema, including scrotal hydroceles [2]. The severe physical disabilities and disfigurement can lead to social stigmatisation, loss of income or livelihoods and impact on mental health. The World Health Organization (WHO) Global Programme to Eliminate LF and the Pacific Programme for the Elimination of LF have led decades of evidence-informed interventions aimed at eliminating the disease as a public health problem through breaking the transmission cycle and managing morbidity and disability in those already affected [1,3].

As most infected individuals do not experience symptoms and therefore will not seek treatment, multiple rounds of mass drug administration (MDA) to whole populations are the primary strategic intervention for breaking the transmission cycle. Historically, MDA was implemented with a two-drug regimen of either diethylcarbamazine (DEC) and albendazole, or in areas without endemic onchocerciasis or loiasis, DEC and ivermectin [4]. These combinations have enabled many countries to successfully achieve the WHO criteria for declaring validation of elimination as a public health problem and move to post-validation surveillance [5]. Despite these programmatic successes, many countries – notably eight in the Pacific Region [6] – are yet to achieve validation.

In 2017, WHO formally recommended a triple-drug regimen of ivermectin, DEC and albendazole (IDA) for countries where two-drug MDA has been ineffective for interrupting transmission [7]. There is evidence that single doses of any of the three anti-filarial drugs were very effective at reducing microfilaraemia for extended periods (up to 12 months for DEC, ivermectin and albendazole) [4,8]. Ottesen and colleagues [8] also stated that ‘All three drugs have different modes of action and while none is completely effective in killing adult worms or inhibiting microfilaria (Mf) production by them, still microfilaraemia can be reduced for very long periods (one year or more) by these single-dose, two-drug treatment regimens’. It would therefore be expected that triple-drug regimens would further enhance the efficacy and longevity of this approach.

The first trials of IDA in Papua New Guinea (PNG) confirmed the above expectations: all 12 of the participants in a study conducted by Thomsen et al. [9] were Mf-negative one year after a single dose; and the six participants followed up for two years remained Mf-negative. In subsequent larger trials in PNG, a single dose of IDA cleared Mf in 96% of participants at one year [10], with 97% of these remaining Mf-negative for up to five years [11]. The findings were further supported by a second trial showing ten-fold reduction in population prevalence of Mf in 2382 participants in 12 villages at one year after a single round of triple-drug MDA [12]. However, a trial in Fiji between 2017 and 2019 gave contrasting results, with Mf clearance in only 65.2% of 72 participants at one year after a single dose of directly observed IDA [13]. Another study of 13 Mf-positive individuals in Samoa who received observed treatment in 2019 found that IDA was effective in clearing Mf within 30 days, and most often within seven days [14]. While there is good evidence on the short-term effectiveness of IDA for clearing Mf in Samoa [14], evidence on the long-term effectiveness, or in other settings with diurnal sub-periodic transmission with an *Aedes* vector, remains sparse. This study aims to evaluate the sustained effectiveness of IDA in clearing Mf in Samoa after ten months to 4.5 years post-treatment, where clearance is defined as the reduction of Mf in the peripheral blood to levels not detectable by microscopy.

## Methods

### Study area and background

Samoa is in the Polynesian region of the Pacific and is home to approximately 200,000 people [15]. The majority of residents live on the two main islands of Upolu – where the capital Apia is located – and the more rural island of Savai'i. Decades of two-drug MDA have failed to interrupt transmission of the *W. bancrofti* parasite [16], which is transmitted primarily by the day-biting *Aedes polynesiensis* mosquito. In 2018, Samoa distributed the first round of nationwide triple-drug MDA, which successfully achieved a self-reported coverage of 80.2% of the total population [17]. A second round planned for 2019 was delayed due to a measles outbreak [18] and the COVID-19 pandemic, and was distributed in September 2023.

### Survey timeline

**Figure 1:**
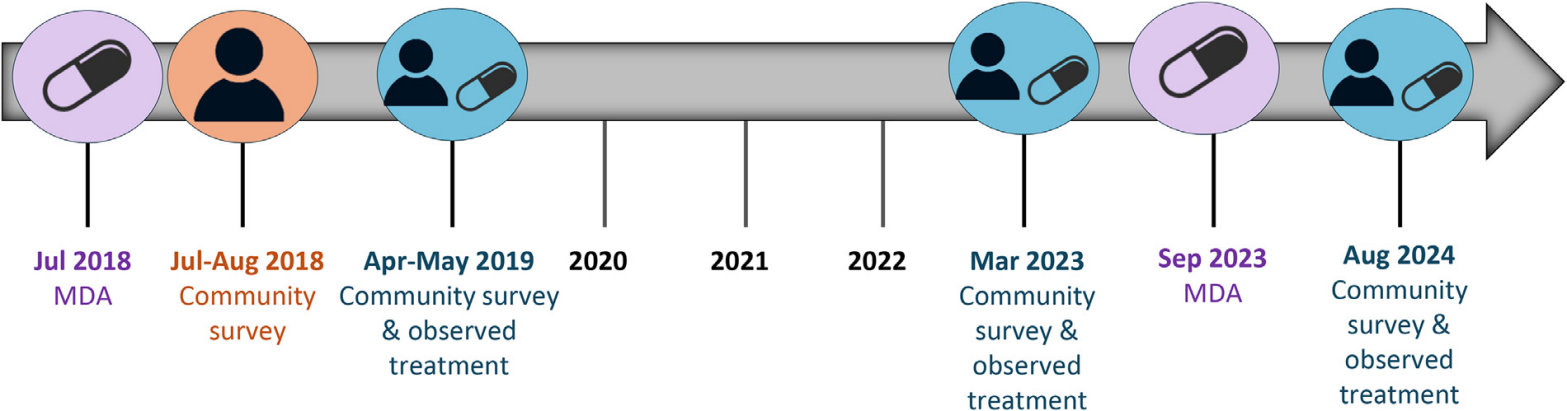
Timeline of Surveillance and Monitoring for the Elimination of Lymphatic Filariasis and Scabies in Samoa (SaMELFS) surveys in Samoa in relation to triple-drug mass drug administration (MDA), 2018-2024.

This study includes longitudinal data from two cohorts, who were participants of surveys carried out by the Surveillance and Monitoring for the Elimination of Lymphatic Filariasis and Scabies in Samoa (SaMELFS) program, which has conducted four community surveys between 2018 and 2024 (Figure 1). The first SaMELFS survey was a nationally representative survey that took place from September 26 to November 9, 2018 [19], commencing 1 month after the completion of the first round of triple-drug MDA. This survey covered 35 primary sampling units (PSUs) across Upolu, Savai'i and Manono Island. The second SaMELFS survey, in 2019, took place from March 28 to May 17, 2019, commencing seven months after the 2018 round of triple-drug MDA in the same 35 PSUs as in 2018 [20,21]. Also in 2019, a study with observed triple-drug treatment was conducted, testing and treating 14 Mf-positive individuals identified in the 2018 survey to assess the effectiveness of IDA for clearing circulating Mf in the short term (1-4 weeks post-treatment) [14]. Of these 14 people, 13 completed follow-up visits and all were confirmed to be Mf-negative within 7-30 days. No treatment was given to other Ag-positive or Mf-positive participants identified in the 2019 survey because an MDA round was planned for shortly after the survey, and Mf results were not available until weeks after the study concluded.

Following a four-year gap in LF elimination activities in Samoa from 2019 to 2022, the next two SaMELFS surveys took place in 2023 from March 2 to April 1 and 2024 from July 25 to August 17. Due to timing and resource limitations, these smaller targeted surveys were only conducted in eight of the 35 PSUs from the previous surveys, and only on Samoa's main island of Upolu [22]. Results of the 2023 survey that was conducted 4.5 years after the first round of triple-drug MDA (and five months prior to the second MDA round in 2023) showed that the first round in 2018 had not been sufficient for breaking transmission, with no significant change in antigen (Ag)-prevalence in eight targeted (non-random) PSUs between 2018 (6.7%, 95% CI 2.9%-12.6%) and 2023 (9.9%, 95% CI 3.5%-21.0%), and high prevalence of Mf-positive cases observed in 2023 (5.1%, 95% CI 1.3%-12.1%) [22]. Surveys in 2023 and 2024 also included a targeted sampling component based on the household location of known Ag-positive participants from previous surveys [23]. Where possible, participants from any component of the 2023 survey who were Mf-positive were treated with IDA within three weeks of being tested, using the same WHO-recommended weight-based dosing used for MDA in Samoa (Supplementary Table S1). In 2024, we attempted to contact and treat as many Ag-positive participants as possible. In both years, a list of people still requiring treatment was provided to the Samoa Ministry of Health.

### Study design

Two cohorts were recruited for this study from among the Mf-positive participants in the previous surveys. Cohort A were tested at five time points, first in July/August 2018 when they were initially identified, and at least twice in 2019 (before and after observed treatment). We then attempted to locate and retest them again in March 2023 and August 2024, along with their household members. The household members were recruited to provide a comparison of Ag and Mf prevalence between cohort members and those living in the same residence. Cohort B were tested twice and consisted of any other Mf-positive participants from the 2023 surveys who received directly observed treatment in that year, and who were tested again as part of the 2024 community survey.

### Data and sample collection

Participants for Cohort A were contacted first via phone, and where this was unsuccessful, through a visit to the person's village. Participants recruited in Cohort B were approached at their place of residence and enrolled using the protocol described in Mayfield et al. [22]. In all cases, demographic data were collected using an electronic questionnaire, and a finger prick blood sample was collected. Samples were tested for LF antigen (Ag) using Alere Filariasis Test Strips (Scarborough, ME, USA). Thick blood smears (slides) of three 20 µL lines were prepared for any Ag-positive samples, according to previously described protocols [19]. Slides were stained with Giemsa according to WHO-recommended methods [24] before being examined under a microscope for Mf. Two slides were read (each by a different reader) for each Ag-positive participant. In 2024, one set of slides was not stained before being read under the microscope.

### Data analysis

Mf densities for Ag-positive participants were calculated as Mf/mL across the two slides, and summarised for different groups using geometric means, adding one if required for comparisons involving zero counts. Change in mean log density between years for each cohort was assessed using unpaired and paired *t*-tests (Stata 17 *t-test* command). Proportions of Ag and Mf positives in cohort and household members were compared using a two-sample test of proportions (STATA 17 *prtest* command).

## Results

### Participants

**Figure 2:**
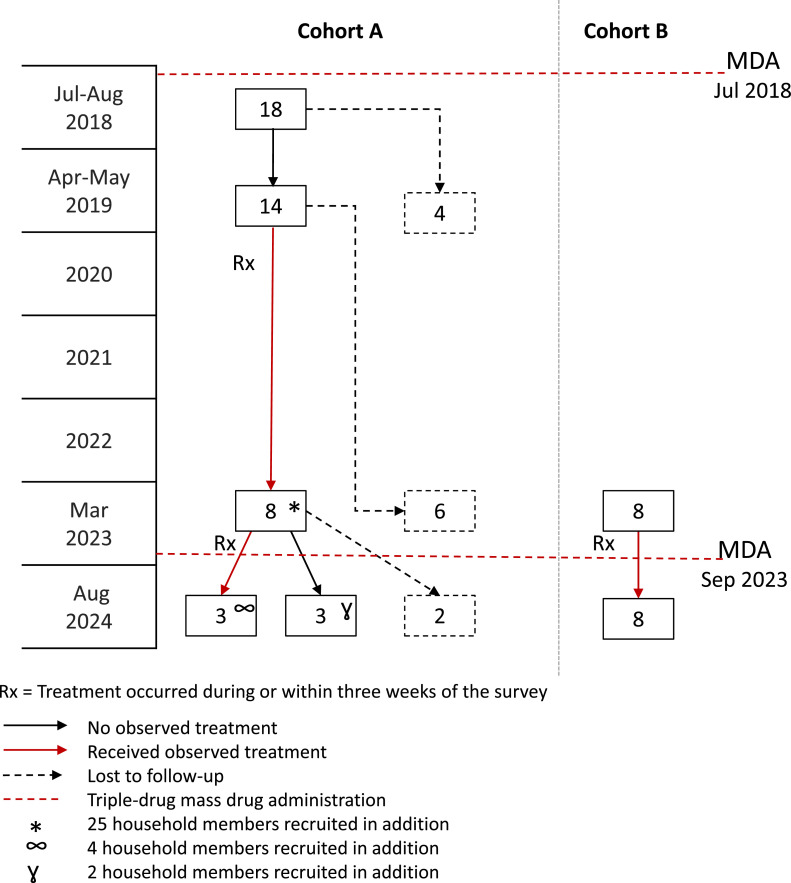
Timeline and number of participants enrolled in the Surveillance and Monitoring for the Elimination of Lymphatic Filariasis and Scabies in Samoa (SaMELFS) 2024 cohort surveys in relation to the triple-drug mass drug administrations (MDA), 2018-2024.

In 2023 and 2024, a total of 16 cohort participants were recruited over the two years (Figure 2). Of the 14 Mf-positive participants from Cohort A who were tested and treated in 2019, eight people from separate households in seven villages (five females and three males, age range 24-64 years) were located in 2023 and agreed to participate. A total of 25 household members were recruited, with at least one household member for each index case (Supplementary Table S2). The mean number of household members per house was 3.1 (min = 1, max = 7). In 2024, six of the eight Cohort A participants from five villages were recruited again, one declined to participate, and one participant was deceased. Six of their household members were also recruited from four households. The mean number of household members per house for these four houses was 1.5 (min = 1, max = 2). For Cohort B, we identified eight Mf-positive participants from the 2023 survey (one female and seven males, age range 49-68 years) who were retested in 2024.

### Antigen and microfilaria prevalence among cohort members

For Cohort A, six of the eight participants (75%) enrolled in 2023 were found to be Ag-positive (Table 1), of whom five were Mf-positive (ID 2, 3, 4, 6, 7), and the sample from the sixth participant (ID 6) having insufficient blood for preparing slides. In 2024, one Ag-negative participant (ID 1) from 2023 was lost to follow-up and the other (ID 5) remained Ag-negative. Of the six Ag-positive participants from 2023, one (ID 3) was lost to follow-up in 2024. Of the five Mf-positive participants treated in 2023 who participated in 2024 (ID 2, 4, 6, 7, 8), two (40%) were Mf-positive (ID 4, 7). For Cohort B, six (ID 9, 10, 12, 13, 15, 16) out of eight participants (75%) remained Ag-positive in 2024, two (33%) of whom were also Mf-positive (ID 9, 15). For the two cohorts combined, of the 13 Mf-positive participants in 2023 who either received treatment and/or reported taking MDA, four (31%) were Mf-positive 18 months later in 2024 (ID 4, 7, 9, 15) (Table 1). All four participants who were Mf-positive in 2024 (ID 4, 7, 9, 15) received observed treatment during the 2023 study. Participant ID numbers corresponding to the direct observed treatment study in 2019 [14] are provided in Supplementary Table S3.

**Table 1:** Demographics antigen (Ag) status, treatment status (directly observed and MDA) and microfilaria (Mf) density (Mf/mL) for participants in Cohorts A and B for each of the four lymphatic filariasis serosurveys in Samoa from 2018 to 2024.

				2018 (July-August)			2019 (April-May)				2023 (March)				2024 (August)	
	ID	Age in 2024	Sex	Ag	Mf density	Took MDA^a^	Ag pre-Rx	Mf density Pre-Rx	Rx	Mf density 7-30 days post-Rx	Ag pre-Rx	Mf density Pre-Rx	Rx	Took MDA^b^	Ag pre-Rx	Mf density pre-Rx
Cohort A	1	50	F	Pos	534.0	No	Pos	784	Yes	0	Neg	0	No	.	.	.
	2	56	F	Pos	33.3	Yes	Pos	66.7	Yes	0	Pos	8.3	No	Yes	Pos	0
	3	48	F	Pos	33.3	No	Pos	241.7	Yes	0	Pos	75	No	.	.	.
	4	64	M	Pos	350.0	Yes	Pos	791.7	Yes	0	Pos	841.7	Yes	Yes	Pos	125.0
	5	57	F	Pos	333.0	Yes	Pos	466.7	Yes	0	Neg	0	No	Yes	Neg	0
	6	24	F	Pos	150.0	No	Pos	25.0	Yes	0	Pos	.	No	Yes	Pos	0
	7	49	M	Pos	75.0	Partial	Pos	66.7	Yes	0	Pos	83.3	Yes	No	Pos	50.0
	8	55	M	Pos	108.0	Yes	Pos	33.3	Yes	0	Pos	50	Yes	Yes	Pos	0

Cohort B	9	64	M	.	.	.	.	.	.	.	Pos	175	Yes	Yes	Pos	75.0
	10	54	M	.	.	.	.	.	.	.	Pos	41.7	Yes	Yes	Pos	0
	11	51	M	.	.	.	.	.	.	.	Pos	41.7	Yes	Yes	Neg	0
	12	65	M	.	.	.	.	.	.	.	Pos	358.3	Yes	Yes	Pos	0
	13	49	M	.	.	.	.	.	.	.	Pos	133.3	Yes	Yes	Pos	0
	14	68	M	.	.	.	.	.	.	.	Pos	191.7	Yes	Yes	Neg	0
	15	52	F	.	.	.	.	.	.	.	Pos	108.3	Yes	Yes	Pos	41.7
	16	56	M	.	.	.	.	.	.	.	Pos	108.3	Yes	Yes	Pos	0

F, female; M, male; Rx, observed treatment with ivermectin, diethylcarbamazine and albendazole (IDA).

Ag-negative participants were assumed to be Mf-negative. Shading represents Ag-positive or Mf-positive test results in that year.

a MDA in 2018, self-reported in 2019.

b MDA in 2023, self-reported in 2024.

### Microfilaria density by cohorts

**Figure 3:**
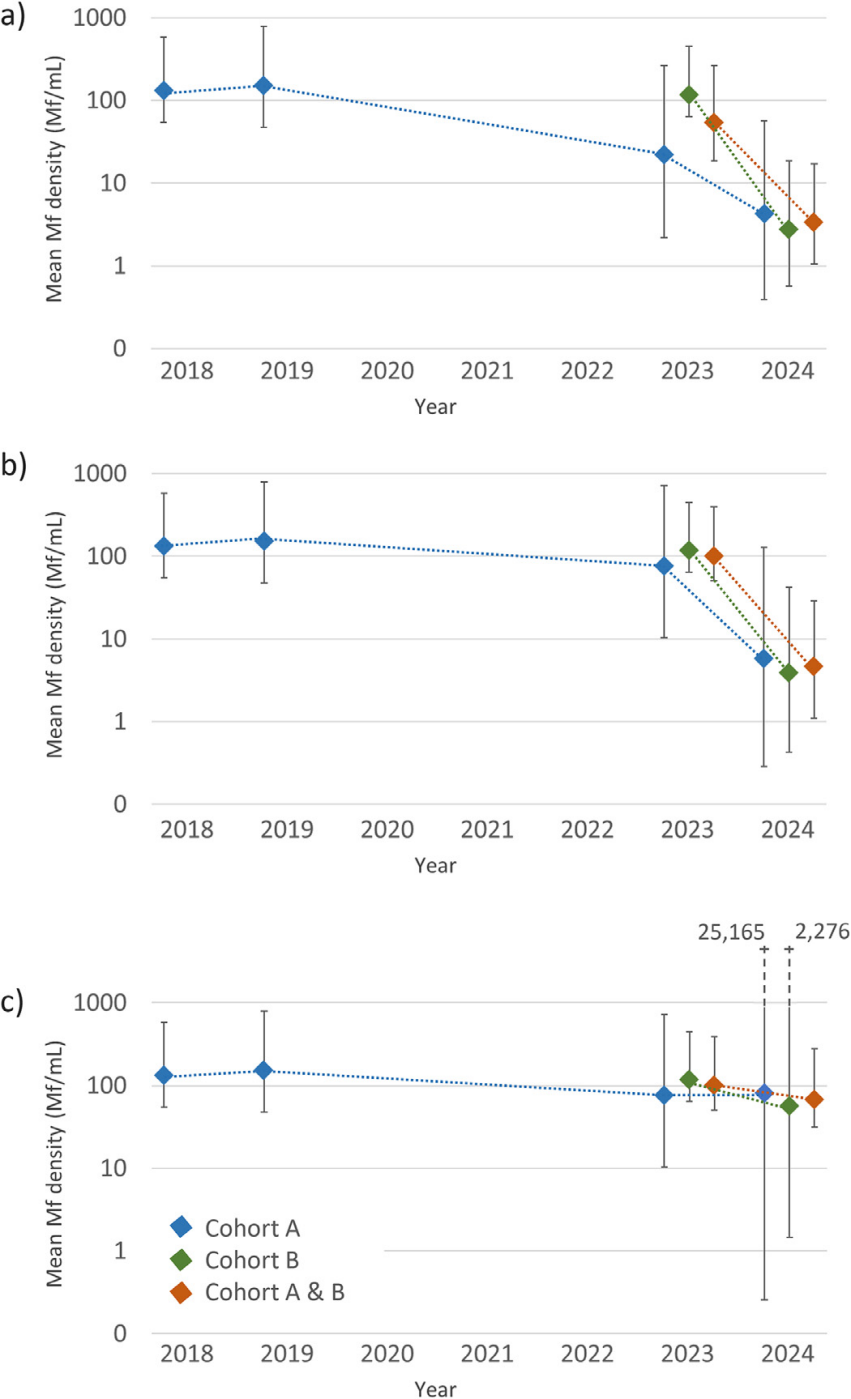
Geometric mean of Mf density for cohort participants in Samoa from 2018 to 2024 for (a) all participants, (b) all Ag-positive participants with valid Mf slides (including Mf-positive and Mf-negative) and (c) all Mf-positive participants. Values are given in Supplementary Table S4.

The geometric mean Mf density for Cohort A, Cohort B and Cohorts A and B combined are given in Figure 3 and Supplementary Table S3. There was a significant decrease in mean log Mf/mL between 2023 and 2024 for all participants (both Ag-positive and Ag-negative) (Figure 3a, *P* < 0.001) and amongst Ag-positive participants (both Mf-positive and Mf-negative) (Figure 3b, *P* < 0.001) because several participants cleared their Mf between 2023 and 2024. Amongst only Mf-positive participants, there was no significant change in mean log Mf/mL between years for any cohort (Figure 3c, *P* = 0.522). However, in the four participants who were Mf-positive in both 2023 and 2024 (two of whom had received observed treatment in both 2019 and 2023, and two only in 2023), there was a significant decrease in the geometric mean density of Mf between years, from 192.3 Mf/mL (95% CI 97.3-992.1 Mf/mL) in 2023 to 67.6 Mf/mL (95% CI 31.5-145.1 Mf/mL) in 2024 (*P* = 0.004).

### Ag and Mf prevalence in household members for Cohort A

In 2023, Ag and Mf prevalence amongst the 25 household members of Cohort A was 28% (95% CI 10.4%-45.6%) and 12% (95% CI 2.5%-31.2%), respectively. Both Ag and Mf prevalence were significantly lower (*P* = 0.018 and *P* = 0.001, respectively) in the household members than for the cohort members, who had Ag and Mf prevalence in 2023 of 75% (95% CI 34.9%-96.8%) and 71% (95% CI 29.0%-96.3%), respectively. In 2024, there were six household members recruited for Cohort A, five of whom were Ag-negative (presumed to be Mf-negative), and one who was Ag-positive, but Mf-negative. A breakdown of counts for Ag-positive and Mf-positive people among household members for each member of Cohort A is given in Supplementary Table S2.

## Discussion

Our study showed that one or two directly observed weight-based doses of triple-drug therapy (IDA) may not be sufficient for sustained clearance of *W. bancrofti* Mf in Samoa. Furthermore, the high self-reported participation rates for the triple-drug MDA in 2018 indicate that some individuals in Cohort A likely had more than two doses of IDA and still did not maintain Mf clearance after 4.5 years. The efficacy of IDA in clearing Mf after a year is also not assured, with 36% of the Mf-positive people who received observed treatment in March 2023 testing Mf-positive again in August 2024, only 18 months later.

The effectiveness of an MDA program to achieve elimination relies on a multitude of factors including program coverage, environmental and behavioural factors, and an individual's response to the medications. A crucial requirement is a drug combination/regimen that results in sustained clearance of Mf so that transmission of the parasite from human to mosquito no longer occurs, i.e. interruption of transmission. In addition to clearance of microfilaraemia, effective treatment regimens should ideally also neutralise adult worms (through death or sterilisation) so that new Mf are not produced and continued damage to the lymphatics does not occur. While all participants in both cohorts received at least one dose of observed IDA treatment, many also reported participating in the MDA rounds in 2018 and 2023, although it is acknowledged that self-reported participation in MDA is not completely reliable.

There are two potential explanations for the persistence of high Mf prevalence in treated participants: recrudescence (reappearance in peripheral blood of Mf from the persistent parent worms), or reinfection (Mf arising from new adult worms matured from L3 larvae originating from subsequent mosquito bites). Our 2019 study in Samoa [14] provided evidence that the triple-drug treatment was effective in reducing Mf levels in the first 7-30 days to below that observable in two 60µL blood smears and one mL of filtered blood. However, five out of eight of these participants were Mf-positive again in 2023, 4.5 years later. This equates to a 70% Mf prevalence among the Cohort A members compared to 12% prevalence in their households. The substantially higher prevalence of Mf in cohort members compared to their household members suggests that recrudescence was more likely than reinfection, especially given both groups reside in the same domestic environment with similar exposures, and the strong clustering of infections at the household level in the Samoan islands [19,25].

Participants from Cohort A and B who were treated in 2023 had an Mf prevalence of 36% in 2024. This is still higher than the prevalence in any community sampled in 2023: of the eight PSUs included in the 2023 community survey, the highest Mf prevalence was 11.9% (95% CI 5.7-20.8) [22], which would be expected to have decreased in 2024 (ten months after the 2023 MDA). Using this population as a comparison, the Mf prevalence amongst the treated cohort participants was substantially higher than expected if their Mf-positivity was the result of reinfection alone.

In contrast to the results from PNG [[9], [10], [11]] which showed 96%-100% clearance of Mf at one year after a single dose of IDA, the results from this study in Samoa are supportive of the findings in Fiji [13], showing that a single dose of IDA was not sufficient for sustained clearance of Mf. Potential explanations for the discrepancies in findings include differences in response to IDA in Polynesian strains of *W. bancrofti* (those with diurnal sub-periodic transmission with an Aedes vector) compared to those circulating in PNG and elsewhere, or differences in human response to treatment in Polynesian populations. Further work is needed to investigate these potential explanations. For example, to definitively test the hypothesis that the observed Mf were caused by persistent infection, the genetic diversity of Mf in a person after IDA treatment administration could be compared with that of Mf taken at an earlier time point to determine whether the Mf from both time points are offspring of the same parent worms (as in Hedtke et al. [26], Choi et al. [27]).

There are several limitations to this study. First, the 4.5-year gap between the 2019 and 2023 surveys created difficulties in locating the original participants, resulting in a small sample size for Cohort A who were tested in all four surveys. Despite the inclusion of Cohort B to provide additional evidence on the effects of a single round of treatment, it remains difficult to reach robust conclusions. Second, while the conclusions that have been made are based on the clearance or persistence of Ag and Mf following observed treatment, the potential additional impact of study participants taking the triple-drug MDA has also been considered. It is acknowledged that MDA participation in each year was self-reported the year after the MDA occurred, and it is possible that not all the study participants who reported taking the medication during the MDA would have actually ingested the pills. Nevertheless, the results present compelling evidence of the need to follow-up infected individuals with additional treatment rounds.

The policy recommendations stemming from these findings are directly relevant to LF elimination programs in Samoa and the broader Pacific region. One or two doses of IDA may not be effective for sustained clearance of Mf, particularly for infections with high Mf density, so Mf-positive individuals need to be followed up post-treatment to prevent progression of lymphatic damage and onward transmission. This is in contrast to current practices which rely on any follow-up treatment being provided during subsequent MDA rounds. This policy is unreliable as MDA rounds can be delayed, or those people needing follow-up treatment may not participate. The findings therefore also demonstrate the importance of completing multiple rounds of MDA without excessive time between rounds, and with high coverage of the at-risk populations. Failure to do so could jeopardise LF elimination efforts aimed at reducing Mf prevalence to below the threshold required to break the parasite transmission cycle and reduce the burden of LF as a public health problem in the Pacific.

## CRediT authorship contribution statement

**Helen J. Mayfield:** Methodology, Formal analysis, Investigation, Data curation, Writing – original draft, Funding acquisition, Project administration. **Ramona Muttucumaru:** Conceptualization, Methodology, Formal analysis, Investigation, Data curation, Writing – original draft, Funding acquisition. **Benn Sartorius:** Formal analysis, Investigation, Writing – original draft. **Sarah Sheridan:** Conceptualization, Investigation, Methodology, Funding acquisition. **Selina Ward:** Data curation, Investigation, Writing – review & editing. **Beatris Mario Martin:** Investigation. **Shannon M. Hedtke:** Investigation. **Robert Thomsen:** Conceptualization, Writing – review & editing. **Satupaitea Viali:** Conceptualization, Funding acquisition, Writing – review & editing. **Glen Fatupaito:** Conceptualization, Writing – review & editing. **Colleen L. Lau:** Conceptualization, Methodology, Formal analysis, Investigation, Writing – original draft, Funding acquisition, Project administration. **Patricia M. Graves:** Conceptualization, Methodology, Formal analysis, Investigation, Data curation, Writing – original draft, Funding acquisition.

## Declarations of competing interest

The authors declare that they have no known competing financial interests or personal relationships that could have appeared to influence the work reported in this article.

## Appendix Supplementary materials

Image, application 1
