# Supplementary material for: Recurrence of microfilaraemia after triple-drug therapy for lymphatic filariasis in Samoa: Recrudescence or reinfection?
Source: Int J Infect Dis. 2025 Mar;152:None. doi: 10.1016/j.ijid.2025.107809 (PMC11873681; doi:10.1016/j.ijid.2025.107809)
Supplement: Supplementary file 1 [file mmc1.pdf]

Recurrence of microfilaraemia after triple-drug therapy for lymphatic filariasis in Samoa: recrudescence or reinfection?

Helen J Mayfield, Ramona Muttucumaru, Benn Sartorius, Sarah Sheridan, Selina Ward, Beatris Mario Martin, Shannon Hedtke, Robert Thomsen, Satupaitea Viali, Glen Fatupaito, Colleen L Lau, Patricia M. Graves

Supplementary

Supplementary Table S1: Weight-based dosing schedule for triple-drug mass drug administration (MDA) in Samoa, 2018-2024.

Weight Range (kg)	Number of Ivermectin Tablets (3 mg)	Number of DEC Tablets (100 mg)	Number of Albendazole Tablets (400 mg)	Total Number of Tablets
<15 kg*	0	1	1	2
15–23 kg	1	1	1	3
24–38 kg	2	2	1	5
39–53 kg	3	3	1	7
54–68 kg	4	4	1	9
69–83 kg	5	5	1	11
84–98 kg	6	6	1	13
99–124 kg	7	7	1	15
>124 kg	8	8	1	17

*Or 2–4 years old)

Source: Samoa Ministry of Health. kg: kilogram; mg: milligram; DEC: diethylcarbamazine.

Supplementary Table S2: Counts of antigen- (Ag) and microfilaria (Mf)- positive household members for Cohort A in 2023 and 2024 Samoa surveys. Shading represents a positive Ag or Mf result.

ID	Ag 2023 Pre-Rx	Mf 2023 Pre-Rx	Rx in 2023	Took MDA in 2023**	Household members in 2023			Ag 2023 Pre-Rx	Mf 2024 Pre-Rx	Household members in 2024		
					n	Ag+	Mf+			n	Ag+	Mf+
1	Neg	Neg	No	.	2	0	0	.	.	0	.	.
2	Pos	Pos	No	Yes	2	0	0	Pos	Neg	2	0	0
3	Pos	Pos	No	.	2	1	0	.	.	0	.	.
4	Pos	Pos	Yes	Yes	2	0	0	Pos	Pos	1	0	0
5	Neg	Neg	No	Yes	7	2	2	Neg	Neg	0	.	.
6	Pos	.	No	Yes	6	2	1	Pos	Neg	0	.	.
7	Pos	Pos	Yes	No	3	1	0	Pos	Pos	1	1	0
8	Pos	Pos	Yes	Yes	1	1	0	Pos	Neg	2	0	0

Shading indicates Ag- or Mf-positive.

Supplementary Table S3: Participant IDs for the eight participants included in Cohort A in the 2023 and 2024 lymphatic filariasis cohort study in Samoa, matched with their corresponding participant IDs from the 2019 observed treatment study reported in Graves *et. al.* 2022 (S1).

**2024 Participant ID**	**Participant ID from Graves *et al* (S1]**
1	2
2	3
3	4
4	5
5	6
6	7
7	9
8	14

Reference S1: Graves, P.M., S. Sheridan, J. Scott, F. Amosa-Lei Sam, T. Naseri, R. Thomsen, C.L. King, and C.L. Lau, Triple-Drug Treatment Is Effective for Lymphatic Filariasis Microfilaria Clearance in Samoa. Tropical Medicine and Infectious Disease, 2021. 6(2): p. 44.

Supplementary Table S4: Geometric mean density (GMD) of Mf/mL for cohort participants in Samoa from 2018 to 2024 for a) All participants, b) All Ag-positive participants with valid Mf slides, and c) All Mf-positive participants. GMD Mf/mL +1 indicate that + 1 has been added to the GMD to allow values to be displayed on a log scale.

		2018				2019				2023				2024			
		n	GMD Mf/mL +1	95% CI lower	95% CI upper	n	GMD Mf/mL+ 1	95% CI lower	95% CI upper	n	GMD Mf/mL +1	95% CI lower	95% CI upper	n	GMD Mf/mL +1	95% CI lower	95% CI upper
Cohort A	All participants	8	132.0	54.7	318.5	8	152.3	47.6	487.2	7	22.1	2.2	220.8	6	4.3	0.4	47.3
	All Ag+ with Mf result	8	132.0	54.7	318.5	8	152.3	47.6	487.2	5	76.2	10.3	562.3	5	5.8	0.3	116.9
	All Mf+	8	132.0	54.7	318.5	8	152.3	47.6	487.2	5	76.2	10.3	562.3	2	80.2	0.3	25088.3
Cohort B	All Ag participants									8	117.2	63.6	215.9	8	2.7	0.6	13.2
	All Ag+ with Mf result									8	117.2	63.6	215.9	6	3.8	0.4	34.7
	All Mf+									8	117.2	63.6	215.9	2	57.0	1.5	2220.0
Cohort A & B	All Ag participants									15	53.8	18.6	155.3	14	3.3	1.1	10.5
	All Ag+ with Mf result									13	99.3	50.9	193.7	11	4.6	1.1	19.5
	All Mf+									13	99.3	50.9	193.7	4	67.6	31.5	145.1
